# Breath-Hold Induced Cerebrovascular Reactivity Measurements Using Optimized Pseudocontinuous Arterial Spin Labeling

**DOI:** 10.3389/fphys.2021.621720

**Published:** 2021-02-17

**Authors:** Sergio M. Solis-Barquero, Rebeca Echeverria-Chasco, Marta Calvo-Imirizaldu, Elena Cacho-Asenjo, Antonio Martinez-Simon, Marta Vidorreta, Pablo D. Dominguez, Reyes García de Eulate, Miguel Fernandez-Martinez, María A. Fernández-Seara

**Affiliations:** ^1^Department of Radiology, Clínica Universidad de Navarra, Pamplona, Spain; ^2^Instituto de Investigación Sanitaria de Navarra (IdiSNA), Pamplona, Spain; ^3^Department of Anesthesia, Perioperative Medicine and Critical Care, Clínica Universidad de Navarra, Pamplona, Spain; ^4^Siemens Healthineers, Madrid, Spain

**Keywords:** arterial spin labeling, breath-hold, cerebrovascular reactivity, cerebral blood flow, hypercapnia

## Abstract

A pseudocontinuous arterial spin labeling (PCASL) sequence combined with background suppression and single-shot accelerated 3D RARE stack-of-spirals was used to evaluate cerebrovascular reactivity (CVR) induced by breath-holding (BH) in ten healthy volunteers. Four different models designed using the measured change in PETCO2 induced by BH were compared, for CVR quantification. The objective of this comparison was to understand which regressor offered a better physiological model to characterize the cerebral blood flow response under BH. The BH task started with free breathing of 42 s, followed by interleaved end-expiration BHs of 21 s, for ten cycles. The total scan time was 12 min and 20 s. The accelerated readout allowed the acquisition of PCASL data with better temporal resolution than previously used, without compromising the post-labeling delay. Elevated CBF was observed in most cerebral regions under hypercapnia, which was delayed with respect to the BH challenge. Significant statistical differences in CVR were obtained between the different models in GM (*p* < 0.0001), with ramp models yielding higher values than boxcar models and between the two tissues, GM and WM, with higher values in GM, in all the models (*p* < 0.0001). The adjustment of the ramp amplitude during each BH cycle did not improve the results compared with a ramp model with a constant amplitude equal to the mean PETCO2 change during the experiment.

## Introduction

The increase in cerebral blood flow (CBF) induced by a vasoactive agent or task, known as cerebrovascular reactivity (CVR), is an indicator of cerebrovascular health. Experimentally CVR can be measured using different brain imaging methods, such as PET, SPECT, transcranial Doppler ultrasonography, and MRI, in combination with a vasodilatory challenge. Although nuclear medicine methods have traditionally been the standard for the assessment of CVR, MRI techniques have emerged as an attractive, noninvasive alternative.

In MRI measurements of CVR, the blood oxygen-level dependent (BOLD) technique has been typically used (Kastrup et al., 2001; Murphy et al., 2011; Bright and Murphy, 2013; Zhou et al., 2015), because it is highly available and easy to implement. However, BOLD does not provide a direct measurement of CBF changes, because the BOLD signal is dependent on several factors besides CBF, including cerebral blood volume and the cerebral metabolic rate of oxygen. In contrast, arterial spin labeling (ASL) can provide a quantitative measure of CBF, allowing CBF-based CVR measurements to be realized, as originally demonstrated by Noth et al. (2006), using pulsed ASL (PASL).

In PASL a volume of arterial blood is labeled almost instantaneously by the application of an inversion pulse that is followed after a time delay by QUIPSS II type saturation pulses (Wong et al., 1998) to control the duration of the labeled bolus, thus rendering the technique capable of quantifying CBF from a single inversion time acquisition. However, in situations of increased blood flow and higher blood velocities, the bolus duration could be effectively shorter than the time delay from inversion to saturation, leading to an underestimation of flow, and thus potentially an underestimation of CVR. This has motivated prior work on the use of pseudocontinuous arterial spin labeling (PCASL) for CVR assessment (Tancredi et al., 2012). In PCASL, quantification of CBF is potentially more accurate since the duration of the label is defined by the length of the labeling pulse. Additionally, PCASL can provide higher perfusion SNR, albeit with lower temporal resolution, since with typically used labeling duration and post-labeling delay the acquisition time per image [henceforth termed repetition time (TR)] is on the order of 4 s, thus yielding a label and control pair every 8 s. To shorten the acquisition time Tancredi et al. decreased the PLD to less than 1 s, thus achieving a TR of 3 s. However, shortening the PLD increases the sensitivity of CBF quantification to variations in the arterial transit time (ATT), since quantification is only accurate if the PLD is longer than the longest ATT value. In healthy gray matter ATT can vary between 500–1500 ms, while this value is longer in the deep white matter.

Recently, Vidorreta et al. (2017) introduced an optimized PCASL sequence with background suppression and an accelerated 3D RARE stack-of-spirals readout that can obtain whole-brain, high SNR perfusion maps, with isotropic resolution. The acceleration was achieved by implementing parallel imaging along the Head-Foot (HF) phase-encoding direction (i.e., slice-encoding direction), thus reducing significantly the image readout time.

In this work, we have evaluated the use of this sequence to measure CVR induced by breath-holding (BH) (Kastrup et al., 1999). The high SNR of the sequence allowed us to use a shorter labelling duration, which together with the accelerated readout time, made possible achieving a TR of 3 s while maintaining a long PLD of 1.4 s. The sequence was tested in a group of healthy volunteers, using a BH task to induce hypercapnia. This task was chosen because BH periods are necessarily of short duration (on the order of 20 s maximum), thus the use of a short TR is more important for this task than for other hypercapnic manipulations that tend to use longer block designs.

Also, we have compared CVR maps obtained by regressing the CBF signal with four different models designed using the measured change in the PETCO2 induced by the BH. Two of the models had a boxcar shape regressor and the other two had a ramp shape regressor. The amplitude of these regressors was built from the change in PETCO2 at the exhale before and after the BH, considering either the variation in each individual BH or the mean variation across all BH cycles. The objective of this comparison was to understand which regressor offers a better physiological model to characterize the cerebral hemodynamic response under BH.

## Materials and Methods

### Subjects

This study was approved by the Ethics Research Committee of the University of Navarra and all the subjects signed a written informed consent prior to inclusion. Ten healthy volunteers [seven females, mean age = 25.2 years, standard deviation (SD) = 4.3] were included in the study. Standard MRI exclusion criteria were followed. Other exclusion criteria were history of pulmonary or cardiovascular disease. No participant needed to be excluded from the study. Volunteers were asked not to smoke or drink caffeine (Perthen et al., 2008) in the 3 h previous to the MRI examination, not to drink alcohol 10 h before the scan and to sleep well the previous night (Moreton et al., 2016).

### MRI Scanning Protocol

All MRI experiments were conducted on a 3T Siemens MAGNETOM Skyra scanner (Siemens Healthcare GmbH, Erlangen, Germany) with a 32-channel head coil. The imaging protocol included a T1-weighted anatomical 3D MPRAGE sequence (with inversion time (TI) = 950 ms, repetition time (TR) = 1760 ms, echo-time (TE) = 3.1 ms, resolution = 1 mm isotropic, scan time = 5:08 min), a time-of-flight (TOF) angiography sequence and a PCASL sequence. The angiograms acquired with the TOF sequence, depicting the carotid and vertebral arteries were used to position the PCASL labeling plane. The position was selected above the carotid bifurcation and below the V3 segment. The plane was oriented as perpendicular as possible to the carotid and vertebral arteries. The PCASL sequence combined PCASL (labeling time = 1.2 s, post-labeling delay = 1.4 s) with background suppression and single-shot 3D RARE stack-of-spirals readout with through-plane acceleration (1D-GRAPPA), as previously described (Vidorreta et al., 2017). The imaging parameters were as follows: TE = 10.33 ms, TR = 3 s, resolution = 3.75 mm isotropic, in-plane FOV = 240 mm × 240 mm, excitation flip angle (FA) = 90°, refocusing FA = 180°, in-plane reconstructed matrix = 64 × 64, 26 slices acquired with 15% oversampling, slice Partial Fourier = 5/8 and R = 2 acceleration in the HF phase-encoding direction (i.e., slice-encoding direction) to achieve a single-shot acquisition (readout length <300 ms). The in-plane spiral readout consisted of two spiral arms acquired in interleaved fashion along the RARE train, with maximum gradient amplitude = 36 mT/m, slew rate = 120 mT/m/ms, and receiver bandwidth = 400 Hz/px (dwell time = 2.5 us). Background suppression consisted of two adiabatic inversion pulses played during PLD time, with timings optimized to null the static tissue signal to 10% of its equilibrium value at the time of readout. A proton density image was also acquired at the start of the ASL scan without background suppression or labeling pulses, for CBF quantification.

### Breath-Hold Task

During the scanning session volunteers were asked to complete a BH task starting with free breathing of 42 s, followed by interleaved end-expiration BHs of 21 s, for ten cycles. The total scan time was 12 min and 20 s. The duration of the BH was adjusted to the TR of the ASL sequence and adapted from previous recommendations by Murphy et al. (2011). The number of cycles of BH and recovery was adapted from recommendations by Tancredi and Hoge (2013) and Lipp et al. (2015). The time after the BH was longer than the BH period in order to allow the volunteer to get to the arterial CO_2_ baseline (Bright et al., 2011). End-expiration BH was chosen because it has been shown that the duration of the breath-hold can be reduced while still obtaining CBF signal changes, and to avoid variations between participants that are introduced by an inspiration before the BH (Kastrup et al., 1999; Thomason and Glover, 2008; Lipp et al., 2015).

Audible instructions for the task were given. The instructions were recorded in an audio file using the free software Audacity (Audacity^®^ 2.2.1,^1^) with simple cues generated using a free voice generator in Spanish. A trigger sent by the MRI sequence started the audio file using PsychoPy2 (The University of Nottingham,^2^) in the MRI sound system, thus synchronizing the data acquisition with the performance of the task.

An MRI-safe intraoperative patient monitoring system (Expression, Invivo. Florida, United States) placed in the MRI room was used to measure the PETCO2 of the exhaled air during the task. The expired gasses were collected through a nasal cannula. Participants were asked to breathe through their nose before and to exhale the remaining air through their nose after the BH to collect the proper breath by breath PETCO2 measure (Urback et al., 2017). No volunteer reported discomfort from using the nasal cannula. The data from the equipment were manually collected for each BH cycle from a video of the monitoring device display, recorded during the acquisition.

Participants were asked to stay awake during the acquisition, silent, with closed eyes, to avoid an increase in CBF due to visual stimulation (Moreton et al., 2016; Li et al., 2018). Also, they were asked to breathe calmly because it has been reported that respiratory challenges that require the patient to make ventilatory effort result in motion artifacts (Moreton et al., 2016). Breathing rate was continuously monitored by means of respiratory bellows and data were saved for subsequent analysis.

### Data Pre-processing

Data pre-processing was carried out using custom scripts in Matlab (Mathworks, MA, United States) and SPM (version 12, Welcome Trust Center for Neuroimaging, University College London, United Kingdom). ASL images were realigned, coregistered to the anatomical T1 and sinc-interpolated to double the time resolution, followed by subtraction of label and control (Aguirre et al., 2002) to generate perfusion weighted images with a temporal resolution of 3 s. Subsequently, the perfusion weighted images were converted to CBF maps using a single-compartment quantification model (Buxton et al., 1998). In this model, a single T1 value of arterial blood was used for quantification, although apneas are known to decrease the arterial oxygen saturation (Delahoche et al., 2005), resulting in a slight decrease in T1. This small T1 reduction has an almost negligable effect on CBF quantification (as demonstrated in the analysis presented in Supplementary Material Section 1).

GM and WM masks were generated from the anatomical T1 using the segmentation tool in SPM. The WM mask was eroded using a disk (size 5), to avoid partial volume effects. Both masks were binarized using a 0.35 threshold. The cerebellum was manually removed from the GM mask. These masks were used to compute the mean CBF time course for each tissue type.

A CBF map during normal breathing (NB) was calculated averaging the twelve perfusion weighted images acquired during the first period of the paradigm, while the volunteer was breathing normally. The CBF response to BH was delayed with respect to the measured respiratory trace, as reported for BOLD signal changes (Birn et al., 2008), and most studies time shift the BH regressor to fit the response (Kastrup et al., 1999; Magon et al., 2009). In this work, the time delay was calculated separately for gray and white matter by cross-correlation of the mean perfusion signal time course with the respiratory trace, shifted in steps of 0.1 s.

Mean CBF in GM for each period between apneas (named from here on baseline GM CBF) was calculated by averaging the values of images acquired during these periods, after taking into account the time delay.

The respiratory trace was also analyzed to compute the respiration rate and amplitude during the rest periods between apneas. First, respiratory traces corresponding to these periods were extracted from the total signal. Then the Fourier Transform (FT) was applied to each of these traces to compute the signal frequency and amplitude. The signal frequency is equivalent to the respiratory rate and the FT amplitude is proportional to the respiratory amplitude (related to tidal volume).

The ΔPETCO2 (expired PCO2 after BH – expired PCO2 before BH) was calculated from the data collected from videos of the monitoring device display. Most BH periods generated a change in the PETCO2, from the basal value measured before BH. The change was annotated with the corresponding scan time, and the mean of these ten values was calculated. This mean value was used during modeling to adjust the regressor amplitude to the mean PETCO2 change in each participant. In addition, the PETCO2 change for each BH was also used in a different model to create an adjusted regressor in which the amplitude in each BH cycle was modified according to this individual values.

### Statistical Analyses

The following statistical analyses were executed using STATA SE 14 (StataCorp, Texas, United States), after checking for data normality: first, differences between gray and white matter in delay and CBF measured during normal breathing were assessed with related samples Student *t*-tests. Subsequently, changes over time in the baseline variables (CBF, PETCO2, respiratory rate, and respiratory amplitude) were evaluated using one-way repeated measures ANOVA with “rest period” as the within-subject factor. When the ANOVA yielded a significant effect of this factor (*p* < 0.05), *post-hoc* tests of trends were performed using orthogonal polynomial contrasts.

Voxel-wise computations of CVR maps were carried out using SPM12. To that end, CBF images were entered into four different general linear models (GLM), each model containing a BH regressor, the motion parameters and their temporal derivates, and a constant offset, thus fitting the voxel-wise signal to the equation:

(1) $$y\,\left(t\right)=\beta_{0}+\beta_{1}\,x\,\left(t\right)+\beta_{m}\,M\,o\,t\,\left(t\right)$$

where x(t) represented the BH regressor and β_1_ yielded the voxel CVR.

**FIGURE 1:**
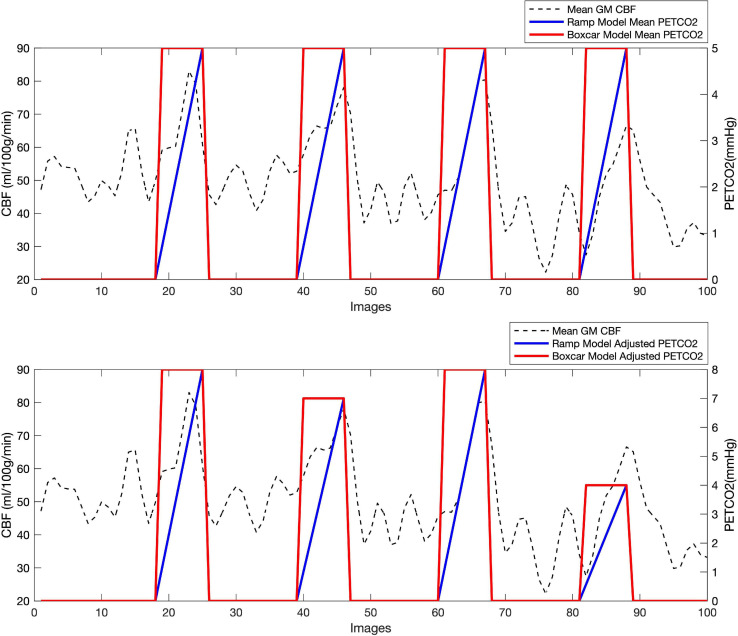
Regressors used in the CVR calculation, shown here for the first 100 images of a representative volunteer: two boxcar regressors (in red), one adjusted in amplitude to a mean PETCO2 change through the whole scan (top), and one adjusted to recorded PETCO2 change in each BH task (bottom) and two ramp regressors (in blue), one varying in amplitude from 0 to the mean ΔPETCO2 recorded in the whole scan (top), and the other from 0 to the recorded ΔPETCO2 for each BH task (bottom). The mean GM CBF (in ml/100 g/min) is also shown for the same subject (discontinuous line).

The BH regressor was different in each model. It was constructed as a boxcar or a ramp of 21 s duration for each BH and adapted to the calculated delay in the GM CBF response. The ratio between the obtained individual delay value and the adjusted TR (3 s) was rounded up to an integer number of images (3 or 4), which corresponded to the correction made to the regressor. The regressor was then scaled by the measured PETCO2 increase (during the BH periods), so that CVR was computed in units of (mL/min/100 g) / mmHg. Four regressors (two boxcar and two ramp models) were obtained by amplitude scaling with a mean ΔPETCO2 change in the whole task, or with the individual ΔPETCO2 in each BH. Representative examples of the four regressors are shown in Figure 1.

The GLM analyses yielded four CVR maps per subject. First, mean CVR values in GM and WM were obtained using the GM and WM masks. A two by four repeated-measures ANOVA (with factors model and tissue) was performed to test for differences between mean GM and WM CVR in the different models. Both factors were found to be significant and thus, *post-hoc* tests were done to assess differences between models and tissues. For these statistical tests, a *p*-value of 0.05, corrected for multiple comparisons using Bonferroni, was considered statistically significant.

Finally, the four CVR maps obtained per subject were normalized to the Montreal Neurological Institute (MNI) template, smoothed (using a 6 mm Gaussian kernel) and compared voxel wise using a second level one-way ANOVA implemented in SPM. Regions of statistically significant differences in CVR, were identified using a significance threshold of 0.05 after correction for multiple comparisons at the cluster level using the family wise error (FEW) correction, with a cluster-generating threshold of *p* < 0.001 (height threshold *T* = 3.33).

## Results

A total of ten volunteers participated in the study. All subjects completed the BH task satisfactorily. There were no images affected by severe artifacts such that they needed to be excluded from the analysis.

### Normal Breathing CBF

The mean value of CBF for GM was 57.2 mL/100 g/min, SD = 14.2, Confidence Interval 95% (CI) = (47, 67.4) and for WM was 36.1 mL/100 g/min, SD = 11, CI = (28.3, 43.9). There were statistically significant differences between the GM and WM mean CBF values (*p* < 0.0001).

### CBF Response to BH

**FIGURE 2:**
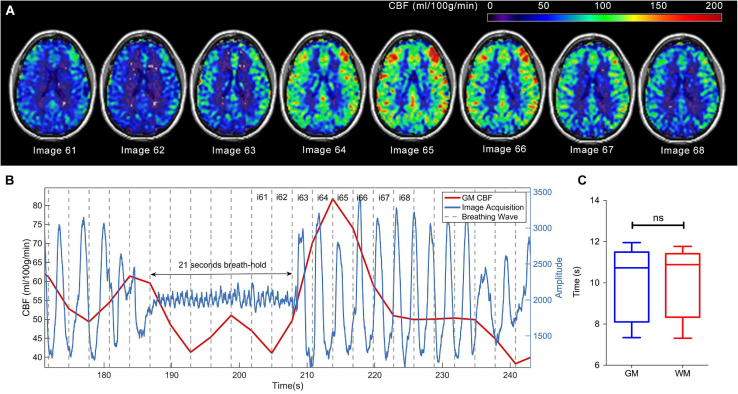
CBF signal increase due to breath-holding in a single participant. **(A)** Consecutive CBF (ml/100 g/min) maps (images 61 to 68) overlayed in anatomical T1-weighted images and shown in neurological orientation. **(B)** Mean CBF (ml/100 g/min) signal in gray matter (red), superimposed on the respiratory trace (blue), where the response delay can be observed. The oscillation in the respiratory trace during the breath-hold period is due to pumping motion of the heart against the chest wall. Gray discontinuous lines represent the period of image acquisition. Maps displayed in **(A)** are indicated by image numbers on top of **(B)**. **(C)** Boxplots showing the measured delays for the GM and WM in the group of volunteers.

As expected, elevated CBF was observed in most cerebral regions under hypercapnia (see Figure 2 and Supplementary Figure 2).

There was a delay in the CBF response to the BH challenge (time between the start of the BH period and the actual CBF increase). The group mean delay was 10.14 s, SD = 1.74, CI = (8.90, 11.38) for GM, and 10.18 s, SD = 1.69, CI = (8.98, 11.39) for WM. There were no significant statistical differences for the delays between GM and WM (*p* = 0.4946). Figure 2 shows a graphic representation of the CBF increase induced by BH in one subject, where the delay can be observed.

**FIGURE 3:**
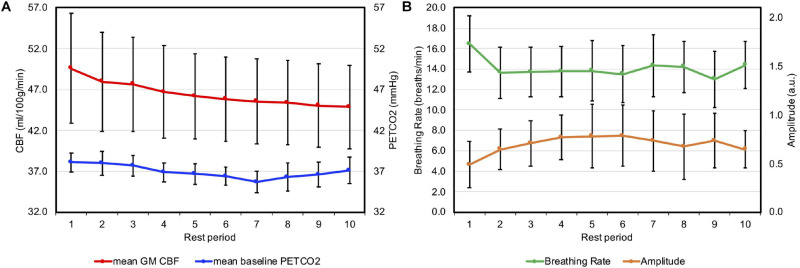
**(A)** Group mean baseline GM CBF (in red and primary axis) and mean baseline PETCO2 (in blue and secondary axis) are presented for the rest periods between apneas, with period 1 being the initial period of normal breathing at the start of the experiment. **(B)** Group mean breathing rate (in green and primary axis) and amplitude (in orange, secondary axis and in arbitrary units) for each rest period. Error bars represent the standard deviation.

Baseline GM CBF values experienced a slight continuous decrease during the experiment (see Figure 3A). This decrease was found to be statistically significant (see Table 1) and contained a linear and quadratic component.

**TABLE 1:** Results of ANOVA and trend analysis for baseline variables.

**Variable**	**ANOVA**		***Post-hoc* test of trends**			
	***F*(9,81)**	***p***	**Lineal**		**Quadratic**	
			***T***	***p***	***T***	***p***
CBF	9.48	0.0049*	−8.78	<0.001	2.59	0.011
CO2	2.56	0.0121	−3.35	0.001	2.89	0.005
Breathing rate		NS				
Breathing amplitude	3.31	0.0018	1.96	0.05	−4.57	<0.001

*corrected with Greenhouse–Geisser epsilon. NS, non significant.

### End-Tidal CO2 and Respiratory Monitoring

In two volunteers, the ΔPETCO2 data were missing for two BH cycles due to technical problems. In these cases, the mean ΔPETCO2 of the whole challenge was used to build the boxcar and ramp for the adjusted ΔPETCO2 regressors. The mean baseline PETCO2 (PETCO2 measure before each BH for all subjects in all BH cycles) was 36.95 mmHg, SD = 0.78, median = 37, interquartile range (IQR) = (35, 39). The group average ΔPETCO2 (for all BH cycles, all subjects) was 4.3 mmHg, SD = 0.48, median = 4.08, IQR = (3.78, 4.73). Individual values of baseline PETCO2 and ΔPETCO2 are reported in the Supplementary Table 1. Baseline PETCO2 values decreased during the experiment (see Figure 3A). This decline was statistically significant (see Table 1) with linear and quadratic components.

Individual values of respiratory rate and amplitude are reported in Supplementary Table 1. Respiratory rates appeared to be stable across the experiment, however, respiratory amplitudes increased after each apnea for the first five cycles (see Figure 3B). This upward trend was statistically significant, with linear and quadratic components (Table 1).

### Breath Holding CVR

**FIGURE 4:**
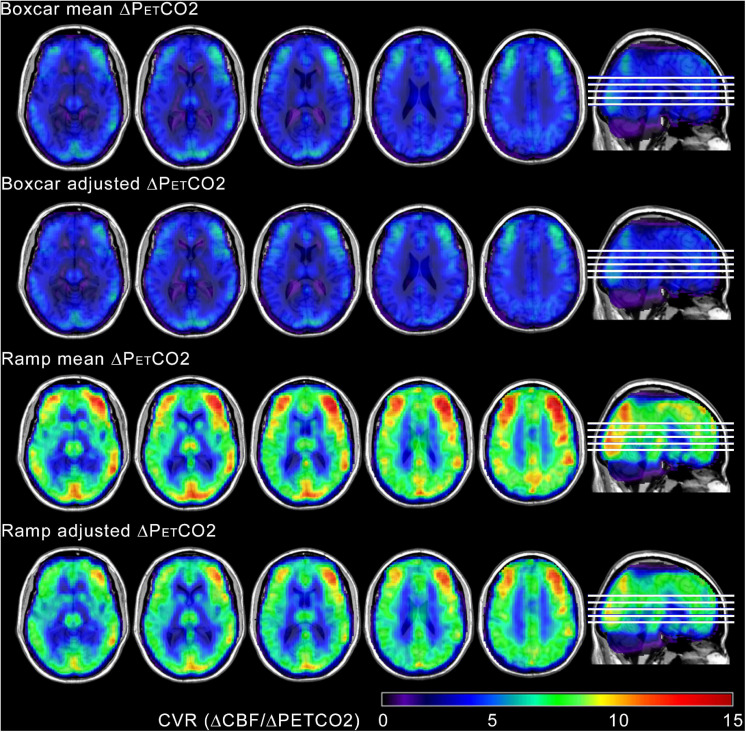
Group mean CVR maps, calculated by averaging normalized individual CVR maps from all ten participants, obtained with the four different tested models. The maps have been overlayed in anatomical T1-weighted template images. The maps display the expected contrast between gray and white matter. Higher values of CVR were obtained using the ramp regressors, with both mean and adjusted ramp amplitudes. Images are shown in neurological orientation.

Figure 4 shows CVR maps normalized and averaged across the whole group computed with the four different models tested. Similarly, individual CVR maps are presented in Supplementary Figure 3. The maps display the expected contrast between gray and white matter and the values are within the range reported in the literature for healthy brain tissue (Tancredi et al., 2012).

**FIGURE 5:**
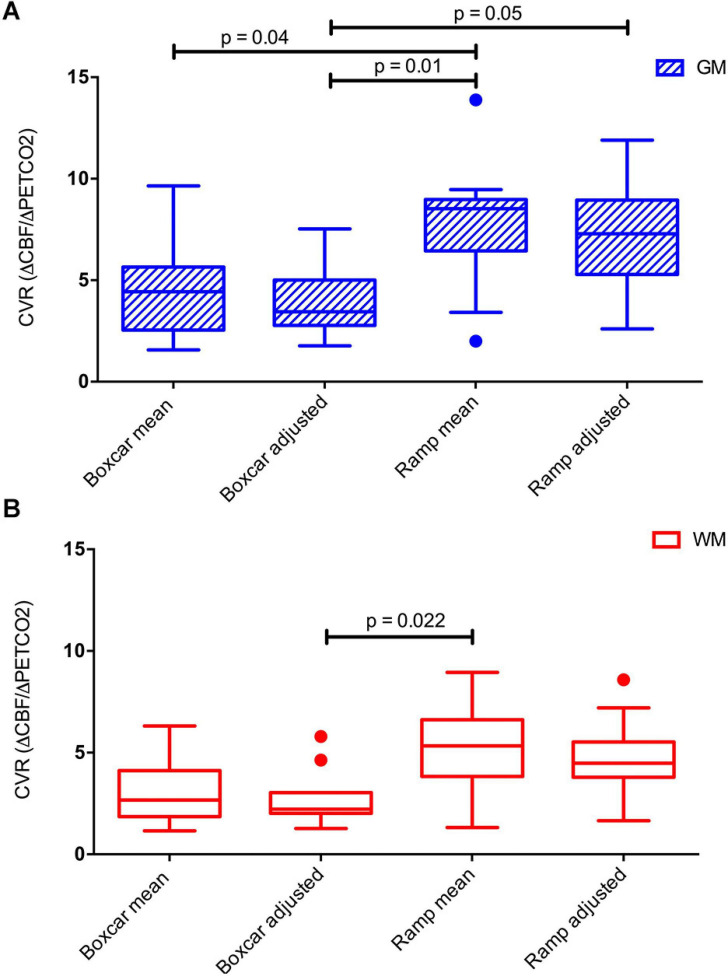
Boxplots of mean GM **(A)** and WM **(B)** CVR computed in the four different models used. Significant differences between the models are indicated. *P*-values have been corrected for multiple comparisons using the Bonferroni method.

At the whole tissue level, statistically significant differences in CVR were obtained among the different models (*p* < 0.0001), with ramp models yielding higher values than boxcar models (see Figure 5) and between the two tissues, GM and WM (*p* = 0.0020), with higher values in GM, in all the models (see Table 2). There were no statistically significant differences between models for the different ways of adjusting the regressor amplitude (i.e., if the regressor was constructed with a mean ΔPETCO2 or with an adjusted ΔPETCO2).

**TABLE 2:** CVR in GM and WM computed with the different models used.

**Brain tissue**	**Boxcar model with mean PETCO2**	**Boxcar model with adjusted PETCO2**	**Ramp model with mean PETCO2**	**Ramp model with adjusted PETCO2**
Mean GM CVR (mL/min/100 g)/mmHg	4.53 (2.34)	3.91 (1.79)	7.88 (3.26)	7.16 (2.81)
Mean WM CVR (mL/min/100 g)/mmHg	3.10 (1.68)	2.69 (1.41)	5.26 (2.25)	4.75 (1.96)
*p*-value (Difference between GM and WM)	<0.0001	<0.0001	<0.0001	<0.0001

*Data are shown as mean (standard deviation). Statistically significant differences were obtained between GM and WM in all models used. *P*-values have been corrected for multiple comparisons using Bonferroni.*

**FIGURE 6:**
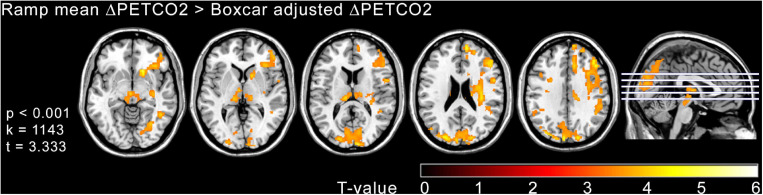
Voxel wise SPM{T} maps obtained in the comparison between the CVR maps estimated with the ramp mean ΔPETCO2 and the boxcar adjusted ΔPETCO2 models (*p* < 0.05 FEW cluster corrected) overlaid on anatomical T1-weighted images. Clusters shown in color scale represent areas of significant CVR increase for the ramp mean ΔPETCO2 model compared to the boxcar adjusted ΔPETCO2 model. Images are shown in neurological orientation.

At the voxel level, statistically significant differences in CVR were only found when comparing the CVR maps estimated with the ramp mean ΔPETCO2 and the boxcar adjusted ΔPETCO2 regressors. Voxel-wise T maps showing areas of CVR differences between these two models are shown in Figure 6. Greater differences are seen in the occipital cortex and frontal gyrus (predominantly on the right side).

## Discussion

In this work, a PCASL sequence combined with background suppression and single-shot 3D RARE stack-of-spirals was used to measure CVR in ten healthy volunteers while doing a BH task. All volunteers were able to complete the task satisfactorily. The accelerated sequence readout allowed to achieve a good temporal resolution, without compromising the postlabeling delay. To compute CVR in (mL/min/100 g) / mmHg, four different models were built using either boxcar or ramp regressors, with amplitude scaled by the change in PETCO2 provoked by the BH. This ΔPETCO2 was a mean over all BHs or an adjusted ΔPETCO2, considering the specific change for each BH. All models assumed that all ten BH challenges lasted 21 s, although the actual duration of the BH might not be the same in all cycles, or in all volunteers.

Ramp models provided higher CVR values, better matching the CBF signal, compared to boxcar models. There were no significant differences between using mean ΔPETCO2 or an adjusted ΔPETCO2. The voxel-wise comparison of CVR maps showed areas in the frontal and occipital lobes with significant CVR differences, between the ramp mean ΔPETCO2 and the boxcar adjusted ΔPETCO2 model. This could mean that these areas are more susceptible to vasodilatation.

There was a delay between the BH and the actual increase in the CBF signal. The mean group delay was 10.14 s, for GM. A previous study using ASL reported a delay of 11 s between the PETCO2 and the mean CBF increases using a gas mixture breathing paradigm (Zhou et al., 2015). Other studies using BOLD and ASL for measuring signal changes provoked by respiratory tasks have reported similar values (≅ 11 to 14 s) of time shift for the regressor used to obtain a good model fit (Birn et al., 2008; Blockley et al., 2011; Bright and Murphy, 2013; Pillai and Mikulis, 2015). WM has been reported to have a longer delay (19 s) compared to GM in healthy subjects (Thomas et al., 2014), with one study reporting that the error in the signal delay is greater in WM (Blockley et al., 2011). In our study there were no significant statistical differences for the delays calculated in GM and WM.

Regarding the origin of this delay, in a BOLD study using gas challenges Zhou et al. pointed out several factors related to the time the gas takes to travel from the pulmonary vascular system to the brain (Zhou et al., 2015). In a breath holding paradigm the increase in the arterial CO2 is gradual, thus there might be other factors causing the observed delay as: the time it takes for the arterial CO2 to travel from the local vasculature in the venous blood to the pulmonary and subsequently to the brain vasculature, the time required for the brain vessel response to the provoked hypercapnia (vasodilatation), and others.

During modeling, this measured delay was corrected by time shifting the BH regressor used in the GLM, but this assumed that the delay was the same for every voxel, which might not reflect what is happening in the brain. It has been suggested that a voxel-specific delay time could be computed in ASL with a voxel-wise map of bolus arrival time (Liu et al., 2017).

There was a significant difference between CBF in GM and WM (*p* < 0.0001) during normal breathing, although the GM – WM contrast was slightly lower than expected. Nonetheless similar CBF values have been reported using ASL in healthy subjects (Fernández-Seara et al., 2005). The lower tissue contrast is likely related to partial volume, which is exacerbated in single-shot 3D readout sequences, especially in the slice encoding direction, due to T2 blurring. Even though the masks were eroded, partial volume is still inevitable, and it likely contributed to the GM CBF underestimation. In addition, the SNR of the CBF measurement during normal breathing was relatively low as it was obtained from the data acquired during the first period of 42 s. A recommendation for obtaining a more accurate measurement of CBF is to do a longer period of rest at the beginning of the scan (Cohen and Wang, 2019).

The pCASL sequence allowed assessing CVR during BH. PCASL was used because it has been reported as the ASL method with the highest reproducibility, precision and SNR (Chen et al., 2011). The implemented sequence had an improved temporal resolution (TR = 3 s) compared to others previously reported (4 to 8 s) (Faraco et al., 2015), due to the shorter readout duration and labeling time, but without compromising the pulse labeling delay, i.e. with a PLD of 1.4 s, which is long enough for the labeled arterial spins to reach the brain tissue in healthy subjects. The reduction in labeling time to 1.2 s from the most commonly used value of 1.5 s was estimated to cause a decrease in perfusion SNR of 14% (see Supplementary Material Section 2). A previous attempt at reducing PCASL TR for CVR measurements used a PLD of 0.9 s (Tancredi et al., 2012). This short PLD value leads to vascular artifacts as the labeled spins do not have enough time to exchange with tissue spins. These bright signal artifacts were not present in our data (see Supplementary Figure 2).

Nonetheless, single time point ASL measurements may be confounded by long arterial transit times (longer than the post-labeling delay). In pathologies where this could be an issue, a multi-delay approach (Teeuwisse et al., 2014) or another labeling strategy such as velocity selective ASL (which is less sensitive to transit time) could be beneficial (Guo and Wong, 2015). However, these strategies suffer from reduced SNR with respect to single time point ASL. Moreover, a multi-delay PCASL acquisition would be difficult to implement in combination with a BH paradigm where minimizing the TR is important to be able to sample the CBF increase generated by the apnea. On the other hand, it could be an interesting alternative to explore when using other types of vasoactive stimuli, that allow for longer block durations.

The end expiration BH task of 21 s period was able to produce PETCO2 changes ranging from 1 to 10 mmHg, with a mean of 4.3 mmHg. This relatively solid change in PETCO2 has been reported to be able to induce a progressive increase in CBF (Kastrup et al., 1999; Bright and Murphy, 2013; Tancredi and Hoge, 2013; Chan et al., 2020). A longer BH could increase the ΔPETCO2, but it might not be suitable for patients or volunteers. The main advantage of this task is that there is no need to use a fixed inspired gas technique, which can generate significant discomfort in the subject. However, the CO2 levels produced during BH are unknown, as there is no CO2 waveform during this period (only values before and after breath holding are available), and it is also unknown the relationship between the time the subject begins breath holding and the subsequent rise in blood CO2 (Pillai and Mikulis, 2015).

An important consideration is that for volunteers to perform end expiration BH they needed to practice outside the MRI room before the scan to be able to complete the task adequately, as it is not normal to hold your breath without air in the lungs. Although volunteers were able to breathe through their nose and do an end expiration BH, this might be a challenge in patients that cannot breathe through their nose or have any other respiratory complications (Liu et al., 2019). Another consideration for future studies is that in order to increase the signal repeatability and robustness it might be better to use paced breathing for rest periods (Scouten and Schwarzbauer, 2008), or to vary the challenge duration during the scan (Bright and Murphy, 2013). These two strategies proposed in BOLD studies, still need to be evaluated in ASL CVR experiments. These considerations are important because variations in the instructions can affect data quality and repeatability (Magon et al., 2009).

As previously mentioned, during this study it was only possible to measure PETCO2 at the beginning and at the end of each BH, to calculate the actual change provoked by the BH task, and this was used to scale the BH regressor. Ideally, a precise continued end tidal CO2 output monitoring system could be used to construct a regressor to better control the PETCO2 baseline (Cohen and Wang, 2019). Importantly, the BH challenge resulted in the expected hypercapnia, but it has been also reported that it can induced mild hypoxia. According to Chan et al., both values, O2 and CO2 should be monitored to assess CVR (Chan et al., 2020), however, Tancredi and Hoge (2013) have demonstrated that there is a minimal vasoactive stimulus provoked by the hypoxia generated in a BH task.

The analysis of the baseline values of PETCO2 and CBF revealed a significant decrease of these two variables across the experiment. This could be caused by rebound hyperventilation post BH, as suggested by the measured increase in breathing amplitude. This finding indicates that an estimation of tidal volume via amplitude analysis with the respiratory rate is a useful tool to determine whether or not the subject is at baseline or steady respiratory status during the rest periods. Conversely, examining respiratory rate alone appears to be inadequate for this purpose, as no significant changes were found through the experiment. Taking these results into consideration, a longer resting period would be necessary to allow PETCO2 and CBF to recover their normal values post BH.

Since the increase of CO2 depends on the metabolic exchange during the BH, we assumed two different models to calculate CVR, with a constant BH duration. The CBF signal seemed to adjust better to the ramp model (yielding higher CVR values), than to a boxcar, suggesting a linear increase in CBF due to BH. However, Murphy et al. (2011) found that a sine regressor adjusted to the BH frequency explained better the PETCO2 variance caused by BH. It would be interesting to consider other nonlinear regressors or models to quantify CVR in ASL in future work.

A limitation of this study is that the temporal resolution of the acquisition was relatively long (3.0 s) compared to BOLD techniques. It would be desirable to use an even shorter TR, but shortening the TR further is difficult to achieve, without compromising the post labeling delay and labeling time. In spite of the lower temporal resolution and lower SNR of ASL with respect to BOLD, there are also several advantages to using ASL for CVR measurements: ASL provides direct measurement of CBF in physiological units, while the BOLD signal has no absolute interpretation and provides only an indirect measurement of CBF changes; ASL can provide measurements of CBF (and therefore CVR) in regions of high static susceptibility gradients, since any pulse sequence can be used as readout, because the signal does not depend on T2^∗^ contrast; the ASL signal is localized to the arterial capillary sites while the BOLD response is dominated by changes in the venous side of the capillary tree, and finally, the ASL signal is stable over long time periods while the BOLD signal suffers from increased noise power at lower frequencies.

Finally, future studies are needed to address the question of whether BH is useful for studying CVR in patients, since BH performance may be more variable and less repeatable. Thus, it would be necessary to control the BH duration and performance with different approaches.

## Conclusion

Pseudocontinuous labeling allowed assessing CVR during BH, with a better time resolution than previously used. The ramp model provided higher CVR values, better matching the CBF signal. There were no significant differences between using mean ΔPETCO2 or an adjusted ΔPETCO2.

## Data Availability Statement

The original contributions presented in the study are included in the article/Supplementary Material, further inquiries can be directed to the corresponding author/s.

## Ethics Statement

The studies involving human participants were reviewed and approved by the Ethics Research Committee of the University of Navarra. The patients/participants provided their written informed consent to participate in this study.

## Author Contributions

SS-B and MF-S all contributed to the design and conceptualization of the study. SS-B, RE-C, EC-A, MC-I, AM-S, MV, PD, RG, MF-M, and MF-S were involved with data collection. SS-B, RE-C, MC-I, MV, MF-M, and MF-S were involved in data analysis. SS-B, RE-C, and MF-S drafted the manuscript. All authors participated in the feedback and writing process following the initial drafting. All authors contributed to the article and approved the submitted version.

## Conflict of Interest

MV was employed by Siemens Healthineers. The remaining authors declare that the research was conducted in the absence of any commercial or financial relationships that could be construed as a potential conflict of interest.

## Footnotes

1 http://www.audacityteam.org/

2 http://www.psychopy.org
