# Supplementary material for: Breath-Hold Induced Cerebrovascular Reactivity Measurements Using Optimized Pseudocontinuous Arterial Spin Labeling
Source: Front Physiol. 2021 Feb 17;12:621720. doi: 10.3389/fphys.2021.621720 (PMC7925895; doi:10.3389/fphys.2021.621720)
Supplement: Supplementary file 1 [file Data_Sheet_1.docx]

Breath-hold induced Cerebrovascular Reactivity measurements using optimized Pseudocontinuous Arterial Spin Labeling

Sergio M. Solis-Barquero, Rebeca Echeverria-Chasco, Marta Calvo-Imirizaldu, Elena Cacho-Asenjo, Antonio Martinez-Simon, Marta Vidorreta, Pablo D. Dominguez, Reyes García de Eulate, Miguel Fernandez-Martinez, María A. Fernández-Seara

Supplementary Material

# Arterial oxygen saturation and blood T1 changes due to breathholding: effect on CBF quantification

Breathholding (BH) can decrease oxygen saturation in arterial blood. Delahoche et al. (Int J Sports Med 26:177-181, 2005) measured a decrease in arterial oxygen saturation (using a pulse oximeter placed on the index finger) in a group of healthy volunteers, performing an apnea of 30 s (at rest) of 5.5%.

The T1 of blood is only weakly dependent on oxygenation, as previously reported (Blockley et al., MRM 60:1313-1320, 2008; Hales et al., JCBFM 36:370-374, 2016). Several studies have measured T1 of arterial and venous blood. In this analysis, the values reported by Lu et al (MRM 52:679-682, 2004) have been employed, as they were measured at 3T. These authors reported values of: T1 = 1664 ± 14 ms (arterial blood, 92% ± 7% oxygenation) and T1 = 1584 ± 5 ms (venous blood, 69% ± 8 % oxygenation). Thus, a decrease of 23% in oxygenation leads to a slight reduction in TR of 80 ms.

These T1 values correspond to R1 values of: R1 = 0.601 s^-1^ (arterial) and R1 = 0.631 s^-1^ (venous).

Assuming a linear change of R1 with oxygenation, a reduction in oxygenation of 5.5% generated by the apnea, will result in an increase in R1 of 0.0072 s^-1^ (i.e. R1 = 0.608 s^-1^).

This yields a T1 = 1644 ms for arterial blood after the apnea.

To calculate CBF the following equation was used, derived from the single compartment model (Buxton et al, MRM, 40:383-96, 1998):

$$CBF= \frac{\Delta M}{M_{0}}\frac{\lambda}{2\alpha}\frac{R_{1\alpha}}{\exp\left( -wR_{1\alpha} \right)-exp(-\left( \tau+w \right)R_{1\alpha})}$$

Where ΔM is the signal difference between label and control images, λ (=0.9ml/g) is the blood/tissue water partition coefficient, R_1a_ is the longitudinal relaxation rate of blood, τ (=1.2 s) is the duration of the labeling pulse, *w* (=1.4 sec) is the post-labeling delay and α (=72%) is the labeling efficiency. M_o_ is the image intensity of the proton density image acquired without background suppression or labeling pulses.

To evaluate the effects of T1 shortening due to apnea on the CBF quantification, this equation was used to compute CBF values for a range of perfusion signal (ΔM/M_0_) from 0.4 to 1 %, using R1 = 0.601 s^-1^ (arterial blood) and R1 = 0.608 s^-1^ (increased after apnea).

The results shown in the graph below (see Supplementary Fig.1) demonstrate that this small increase in R1 (reduction in T1) has a negligible effect on the CBF calculation.

# Reducing the labeling duration: effect on perfusion SNR

The labeling time used in this work was 1.2 s, which is shorter than the labeling time most frequently used of 1.5 s. The loss in SNR due to this shorter labeling duration has been estimated using the following equation, derived from the single compartment model (Buxton et al, MRM, 40:383-96, 1998):

$$\frac{\Delta M}{M_{0}}=CBF \frac{2\alpha}{}\frac{\exp\left( -wR_{1a} \right)-exp(-\left( \tau+w \right)R_{1a})}{R_{1a}}=CBF \frac{2\alpha}{}\frac{\exp\left( -wR_{1a} \right)(1-exp(-\tau R_{1a})}{R_{1a}}$$

Substituting for τ = 1.2 s and τ = 1.5 s and calculating the ratio, we obtained a value of 0.865, which means that the SNR expected with τ = 1.2 s is reduced by 14% with respect to the SNR expected with τ = 1.5 s.

# Supplementary Figures and Tables

## Supplementary Tables

**Supplementary Table 1. PETCO2 (baseline and change) and breathing parameters (rate and amplitude) values in ten volunteers. Values are means across the ten task cycles.**

| **Subject** | **1** | **2** | **3** | **4** | **5** | **6** | **7** | **8** | **9** | **10** |
| --- | --- | --- | --- | --- | --- | --- | --- | --- | --- | --- |
| Baseline PETCO2 (mmHg) | 35.29 (0.45) | 38.73 (1.03) | 47.45 (1.10) | 67.60 (3.74) | 45.63 (3.79) | 48.61 (1.24) | 27.42 (0.62) | 49.04 (2.19) | 50.93 (1.76) | 54.09 (1.85) |
| ΔPETCO2  (mmHg) | 3.9 (0.74) | 4.8 (1.48) | 3.8 (1.62) | 3.7 (1.06) | 5.3 (2.31) | 4.0 (1.41) | 5.9 (1.46) | 4.6 (0.74) | 4.7 (1.80) | 5.2 (1.20) |
| Breathing rate  (breaths/min) | 7.50 (1.18) | 6.75 (0.70) | 13.88 (1.07) | 21.13 (1.25) | 18.05 (2.38) | 14.95 (2.04) | 10.73 (1.60) | 16.37 (1.33) | 21.86 (1.52) | 9.59 (3.70) |
| Breathing Amplitude (a.u.) | 0.73 (0.12) | 1.22 (0.17) | 0.66 (0.31) | 0.42 (0.09) | 0.71 (0.19) | 0.82 (0.25) | 0.83 (0.16) | 0.47 (0.12) | 0.38 (0.05) | 0.72 (0.10) |

Data are shown as mean (standard deviation). Baseline refers to PETCO2 values before each breath hold. ΔPETCO2 refers to the change provoked by each breath hold. The breathing rate and amplitude were measure in the rest self-paced breathing period before each BH. a.u.: arbitrary units.

## Supplementary Figures


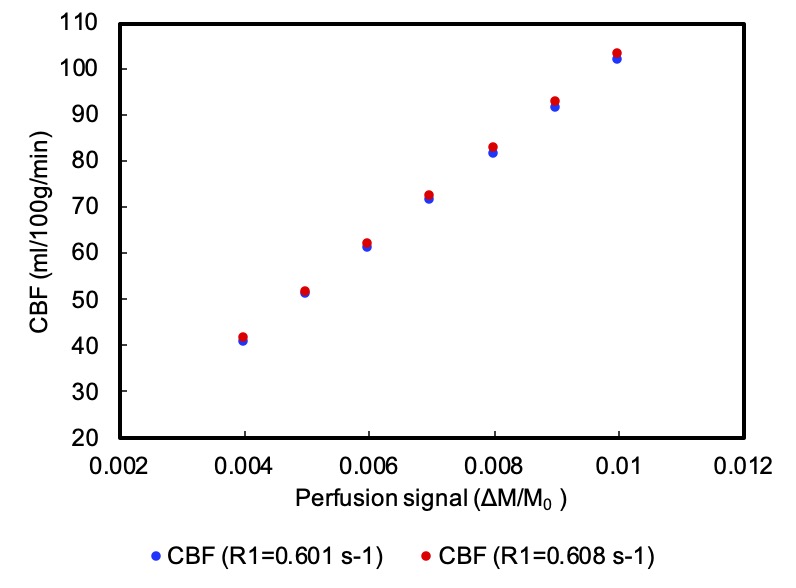


**Supplementary Figure 1.** Computed CBF values for a range of perfusion signal (ΔM/M_0_), considering blood R1 values of 0.601 s^-1^ (T1=1664 ms) (in blue) and 0.608 s^-1^ (T1=1644 ms) (in red).


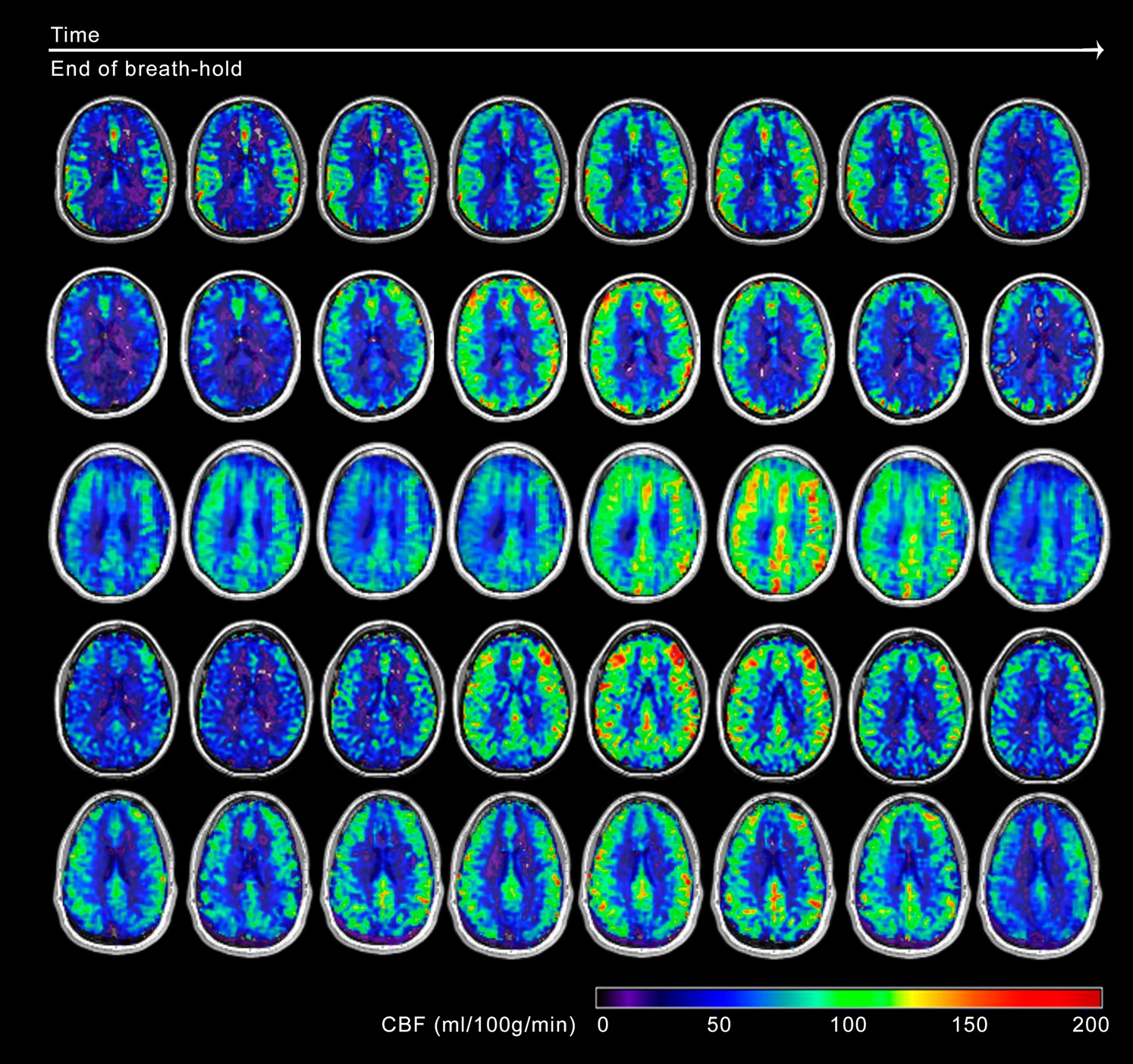


**Supplementary Figure 2.** CBF maps for five volunteers consecutively acquired after one BH period (one subject is presented per row).


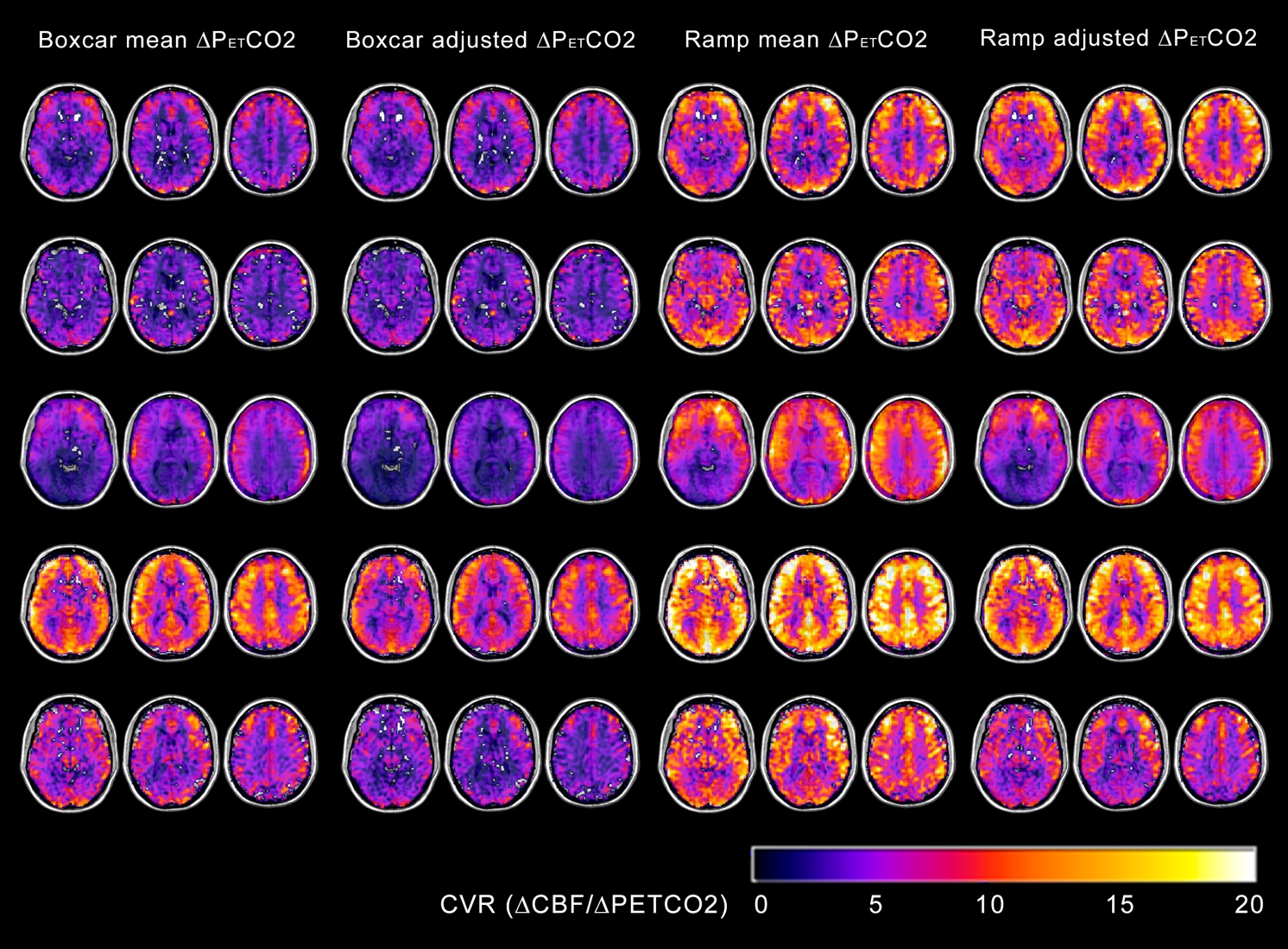


**Supplementary Figure 3.** CVR maps for five volunteers (one subject is presented per row) obtained using the four different tested models.
